# Performance of Existing Definitions and Tests for the Diagnosis of Invasive Fungal Diseases other than Invasive Candidiasis and Invasive Aspergillosis in Critically Ill, Adult Patients: A Systematic Review with Qualitative Evidence Synthesis

**DOI:** 10.3390/jof7030176

**Published:** 2021-02-28

**Authors:** Daniele R. Giacobbe, Andrea Cortegiani, Ilias Karaiskos, Toine Mercier, Sofia Tejada, Maddalena Peghin, Cecilia Grecchi, Chiara Rebuffi, Erika Asperges, Valentina Zuccaro, Luigia Scudeller, Matteo Bassetti, the FUNDICU investigators

**Affiliations:** 1 Department of Health Sciences, University of Genoa, 16132 Genoa, Italy; matteo.bassetti@unige.it; 2 Clinica Malattie Infettive, Ospedale Policlinico San Martino–IRCCS, 16132 Genoa, Italy; 3 Department of Surgical, Oncological and Oral Science (Di.Chir.On.S.), University of Palermo, 90127 Palermo, Italy; cortegiania@gmail.com; 4 Department of Anaesthesia Intensive Care and Emergency, Policlinico Paolo Giaccone, 90127 Palermo, Italy; 5 Hygeia General Hospital, 15123 Athens, Greece; ikaraiskos@hygeia.gr; 6 Department of Microbiology, Immunology and Transplantation, KU Leuven, 3000 Leuven, Belgium; toine.mercier@uzleuven.be; 7 Department of Hematology, University Hospitals Leuven, 3000 Leuven, Belgium; 8 Clinical Research/Epidemiology in Pneumonia & Sepsis (CRIPS), Vall d’Hebron Institute of Research (VHIR), 08035 Barcelona, Spain; stmagraner@gmail.com; 9 Centro de Investigación Biomédica en Red de Enfermedades Respiratorias (CIBERES), Instituto de Salud Carlos III, 28220 Madrid, Spain; 10 Infectious Diseases Division, Department of Medicine, University of Udine and Azienda Sanitaria Universitaria Integrata di Udine, 33100 Udine, Italy; maddalena.peghin@gmail.com; 11 Infectious Diseases Unit, IRCCS San Matteo, 27100 Pavia, Italy; cecigrecchi@gmail.com (C.G.); erika.asperges01@universitadipavia.it (E.A.); zuccaro.v@gmail.com (V.Z.); 12 Department of Internal Medicine and Therapeutics, University of Pavia, 27100 Pavia, Italy; 13 Scientific Direction, IRCCS Istituto Giannina Gaslini, 16147 Genoa, Italy; chiararebuffi@gaslini.org; 14 Scientific Direction, Clinical Epidemiology and Biostatistics IRCCS, Ca’ Granda Ospedale Maggiore Policlinico di Milano Foundation, 20122 Milan, Italy; luigia.scudeller@policlinico.mi.it

**Keywords:** pneumocystis, PJP, diagnosis, biomarker, IFD, invasive fungal diseases

## Abstract

The Fungal Infections Definitions in Intensive Care Unit (ICU) patients (FUNDICU) project aims to provide standard sets of definitions for invasive fungal diseases (IFDs) in critically ill, adult patients, including invasive aspergillosis (IA), invasive candidiasis (IC), *Pneumocystis jirovecii* pneumonia (PJP), and other non-IA, non-IC IFDs. The first step of the project was the conduction of separated systematic reviews of the characteristics and applicability to critically ill, adult patients outside classical populations at risk (hematology patients, solid organ transplant recipients) of available definitions and diagnostic tests for IFDs. We report here the results of two systematic reviews exploring the performance of available definitions and tests, for PJP and for other non-IA, non-IC IFDs. Starting from 2585 and 4584 records for PJP and other IFDs, respectively, 89 and 61 studies were deemed as eligible for full-text evaluation. However, only two studies for PJP and no studies for other IFDs met the FUNDICU protocol criteria for inclusion in qualitative synthesis. Currently, there is no sufficient solid data for directly evaluating the performance of existing definitions and laboratory tests for the diagnosis of PJP and other non-IA, non-IC IFDs in critically ill adult patients outside classical populations at risk.

## 1. Introduction

Invasive candidiasis (IC) and invasive aspergillosis (IA) are the most frequent invasive fungal diseases (IFDs) encountered in non-immunocompromised critically ill patients [1,2,3,4]. The diagnosis of some IFDs (e.g., IA) is difficult in non-neutropenic, non-hematology populations, owing to the frequent absence of proven diagnosis (histology or culture from normally sterile sites), the suboptimal performance of definitions developed for severely immunocompromised patients, and the lack of wide consensus about alternative sets of definitions that have been proposed over the years [5,6,7,8,9].

This lack of standard definitions does not regard only IA and IC, but also other less common IFDs that may be encountered in critically ill patients, such as pneumocystosis and infections due to other yeasts and molds. Against this background, the Fungal Infections Definitions in Intensive Care Unit (ICU) patients (FUNDICU) project was started with the aim of providing standard set of definitions for IFDs in critically ill, adult patients [10]. The first step of the project was the conduction of separated systematic reviews of the characteristics and applicability to critically ill, adult patients of available definitions and diagnostic tests for IFDs: (i) IA; (ii) IC; (iii) other IFDs.

In the present paper, we report the results of the systematic review exploring the performance of available definitions and tests for the diagnosis of IFDs other than IC and IA.

## 2. Methods

The present study follows the Preferred Reporting Items for Systematic Reviews and Meta-Analyses (PRISMA) guidelines [11] and was registered in PROSPERO with number CRD42020170421. The full protocol of the FUNDICU project was published elsewhere prior to study start [10].

### 2.1. Data Sources and Data Management

The PubMed, EMBASE, CINAHL (EBSCOHost), and Cochrane (Wiley) databases were searched with predefined search strings (see Supplementary Materials). The search period was 15 years (from 2003 to 2018). The EndNote Web database was used from importing and managing abstracts and full texts, that were shared among A.C., C.G., D.R.G., I.K., M.P., S.T., T.M., E.A., V.Z., and the librarian (C.R.). Two separated systematic reviews were performed, one for *Pneumocystis jirovecii* pneumonia (PJP) and one for other non-IA, non-IC IFDs. A minor protocol deviation should be acknowledged: no separated systematic review was conducted for cryptococcosis, which was eventually considered within other non-IA, non-IC IFDs [10].

Abstract and full-text review for PJP was independently performed by A.C. and T.M., with disagreements being resolved by a third reviewer (I.K.). Abstract and full-text review for other IFDs was independently performed by E.A., M.P., and S.T., with disagreements being resolved by a fourth reviewer (C.G.).

References of retrieved full texts were also screened to identify further studies suitable for inclusion. Supervision of the review process was provided by D.R.G., V.Z., L.S., and M.B.

### 2.2. Inclusion and Exclusion Criteria

The following studies were considered for inclusion: randomized controlled trials (RCT), single-arm studies, quasi-experimental studies, cross-sectional studies, and prospective or retrospective cohort studies, which assessed the diagnostic performance of definitions and/or tests vs. (preferably) a reference standard (e.g., histology or culture from normally sterile sites) or a reference definition.

Studies were excluded if (i) they were conducted exclusively in the pediatric population (younger than 18 years old); (ii) the performance of definitions/tests for the diagnosis of a specific IFD could not be separated from the performance for other IFDs considered in the given study; (iii) specific reference categories (e.g., possible IFDs) were excluded from the assessment of the diagnostic performance of the evaluated definition/s or tests/s; (iv) they were conducted in a critically ill population exclusively composed of hematology and/or solid organ transplant (SOT) patients; (v) for PJP, they were conducted in a population composed of ≥50% human immunodeficiency virus (HIV)-positive patients (who represent the classical population for PJP development). The last two exclusion criteria are protocol amendments (they were not defined in the initial protocol), which were deemed necessary in order both not to include studies conducted in classical populations for which dedicated definitions/experience already exist and not to exclude studies with mixed (classical and nonclassical) populations from which some information pertaining to nonclassical populations could be extracted.

### 2.3. Data Extraction

Data were extracted on a standard form as previously described [9,10]. The following information was collected for each study: first author; year of publication; study type (RCT, observational); study timeline (cross-sectional, retrospective, prospective); site of IFD; study population; ward/s; number of patients; reference definition/test; reference diagnostic categories (e.g., IFD/non-IFD, non-IFD/possible IFD/probable IFD/proven IFD); number of patients stratified according to the different reference categories; definitions/tests evaluated in the study; diagnostic cutoffs (if applicable); diagnostic performance of the evaluated tests/definitions against the reference (sensitivity; specificity; negative predictive value (NPV); positive predictive value (PPV); positive likelihood ratio (LR+); negative likelihood ratio (LR−); diagnostic odds ratio (DOR)).

### 2.4. Risk of Bias

The risk of bias was quantified with a scoring tool specifically designed for the FUNDICU project [10], with one point being assigned for each of the following eight possible sources of bias (whenever applicable) and a higher overall score indicating lower quality:

- retrospective design and data collection;
- missing classification for >10% of included patients;
- study population also including hematology/SOT patients (and HIV-positive patients for PJP);
- exclusion of patients with difficult-to-diagnose IFDs;
- mixed population of children and adults with ad hoc selection of the diagnostic cutoff;
- unreliable reference (i.e., any reference standard different from histology or culture from normally sterile sites, or microscopy for PJP);
- classification as IFD after knowledge of the result of the reference standard.

### 2.5. Quantitative Data Synthesis

Quantitative data synthesis was not applicable for the purposes of the present project [10].

## 3. Results

### 3.1. Pneumocystis jirovecii Pneumonia

**Figure 1:**
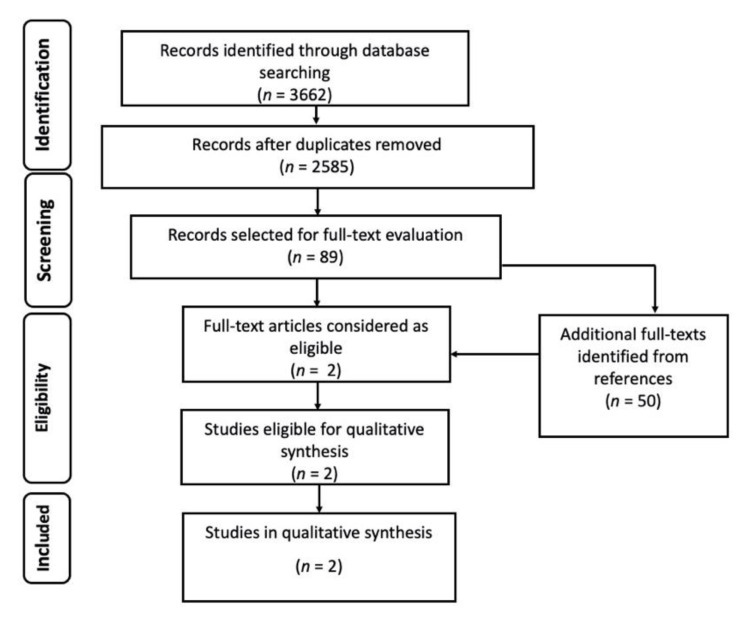
Flow diagram of the study selection process for *Pneumocystis jirovecii* pneumonia (PJP).

The initial number of records was 3662, from which we removed 1077 duplicates. After abstract and title screening of the remaining 2585 records, 89 studies (plus 50 retrieved from references) were deemed eligible for full-text evaluation, and only two of them were ultimately retained for qualitative synthesis (Figure 1). Both were single center studies; one was a prospective cohort study and the other one a retrospective cross-sectional study. Both evaluated the diagnostic performance of polymerase chain reaction (PCR) tests against a reference test, conventional stain plus immunofluorescent assay [12], or microscopic examination [13] for the identification of *Pneumocystis jirovecii*. Both studies included patients with clinical and/or radiological signs of pneumonia, admitted to the ICU or other wards (table 1). In the study by Azoulay and colleagues, the patient population was composed of non-HIV immunocompromised patients, including SOT patients, hematology patients, and patients with solid cancer, with rheumatic diseases, or receiving immunosuppressive drugs for other purposes. The study by Rohner and colleagues included SOT patients, HIV patients, patients with cancer and other forms of immunosuppression, and patients with chronic respiratory diseases. The diagnostic performance of PCR in comparison to the reference tests used in the study is reported in Table 1. Both studies demonstrated a high sensitivity (87.2%; 100%) and specificity (92.2%; 92.4%), as well as a high negative predictive value (NPV, 98.7%; 100%), but a relatively low positive predictive value (PPV, 51.5%; 63.4%).

**Figure 2:**
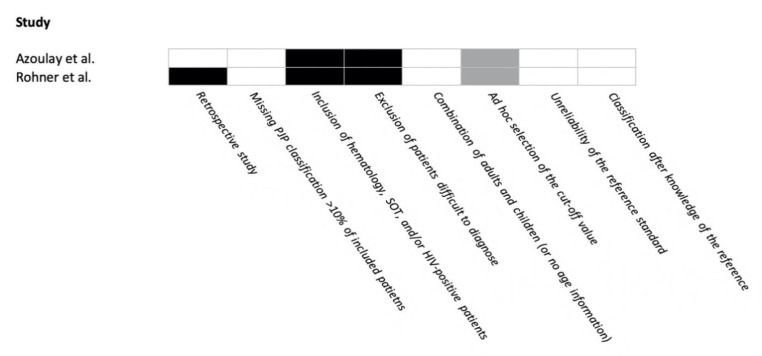
Risk of bias in included studies for *Pneumocystis jirovecii* pneumonia. Risk of bias: present = black; absent = white; not applicable = gray.

The risk of bias in the included studies is reported in Figure 2. One study had <3 points and the other had 3 points in the FUNDICU quality scoring system.

### 3.2. Other Non-IA, Non-IC IFDs

**Figure 3:**
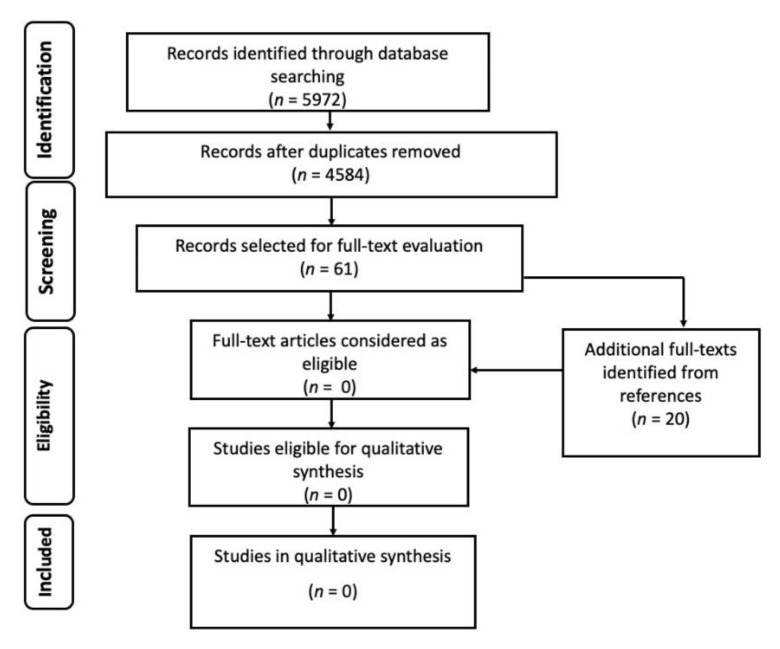
Flow diagram of the study selection process for other invasive fungal diseases.

The initial number of records was 5972, from which we removed 1388 duplicates. After abstract and title screening of the remaining 4584 records, 61 studies (plus 20 retrieved from references) were deemed eligible for full-text evaluation, but none of them met the minimum standards required by the FUNDICU protocol for inclusion in qualitative data synthesis (Figure 3).

## 4. Discussion

Data regarding the diagnostic performance of tests or existing definitions for IFDs other than IA and IC in critically ill adult patients are scant, likely reflecting their lower incidence compared with IC and IA in this specific setting. Nonetheless, these IFDs should not be overlooked from a clinical perspective. For example, once mostly limited to HIV-positive patients with acquired immunodeficiency syndrome and subsequently described in hematological and SOT populations, severe PJP pneumonia requiring intensive care may also develop in patients with other forms of immunosuppression, such as in patients with other types of T-cell deficiency, concurrent chemoradiotherapy, malnutrition, or prolonged high-dose steroid therapy [14,15,16,17]. One of the two aims of the present systematic review was to provide a qualitative summary of the available moderate- to high-quality evidence about the performance of existing tests and definitions for the diagnosis of PJP in HIV-negative, non-hematological, non-SOT critically ill adult patients. Following the rigorous criteria of the FUNDICU protocol [10], only two studies were included, which compared PCR vs. two different references in populations including critically ill patients. They showed a high NPV, but a suboptimal PPV, which overall may make PCR unsuitable as a standalone diagnostic tool in the case of positive results, especially when reasonable differential diagnoses exist and/or the prevalence of the disease is low (as expected for PJP outside of classical populations). Notably, no eligible studies compared qualitative and quantitative PCR results (with the latter possibly improving PPV by setting a cutoff in order to recognize colonization with low fungal burden, especially in the presence of alternative diagnoses) [18,19,20,21], and there were also no eligible studies addressing the diagnostic performance (especially its PPV in view of possible false positive results and the low prevalence of PJP outside of classical populations) of serum beta-d-glucan, which is a useful diagnostic support for PJP in classical populations [22]. Altogether, the most important result of the FUNDICU search (albeit a “negative” one) is the lack of evidence about the diagnostic performance for PJP of available tests/definitions directly in the population targeted by the project (non-HIV, non-hematological, non-SOT critically ill adult patients). In this regard, the next step will be to discuss within the panel whether or not to develop dedicated definitions of PJP for critically ill patients with specific risk factors (different degrees and types of nonclassical immunosuppression) on the basis of studies or existing definitions conducted in/developed for noncritically ill, classical populations at risk of PJP. Whatever the decision of the panel, a detailed account of the motivations will be provided in the final consensus document. In this regard, it is important to note that we decided not to discuss in the present manuscript suggestions (e.g., role of different types and cutoffs of quantitative PCR) not subjected to the strict inclusion criteria of this first phase of the FUNDICU project. Indeed, their interpretation would not be systematic, a fact that may provide arbitrary indications without the proper framework (structured panel discussion with formal vote, as will be the case in the next part of the project). In our opinion, it would be crucial in the future to launch large projects aimed at understanding how tests are used in nonclassical patients tested for PJP (i.e., who are those critically ill suspected to have PJP outside classical populations? Can different phenotypes be identified according to baseline disease/lack of alternative diagnoses? Is there wide heterogeneity in the types of populations tested across centers/regions?). The ultimate aim of this additional baseline knowledge would be to provide more solid guidance for any subsequent assessment of the diagnostic performance of tests in specific subgroups/phenotypes.The second aim of the present systematic review was to provide a qualitative summary of the available moderate- to high-quality evidence on the performance of existing tests and definitions for the diagnosis of other non-IA, non-IC, non-PJP IFDs in non-hematological, non-SOT critically ill patients. As largely expected, likely owing to the rarity of other IFD diseases (although cutaneous mucormycosis in trauma and burn patients is a possible rapidly evolving complication that should be recognized [23,24,25]), no diagnostic studies met the inclusion criteria of the FUNDICU protocol for inclusion in qualitative synthesis [10]. Notably, while the results of the present search will likely discourage the development of dedicated definitions for critically ill adult patients of IFDs other than IA and IC (and possibly PJP) within the FUNDICU project, they may open the door to future, concerted, multinational research efforts to solidly fill the current gap in the knowledge of the best diagnostic approach to rare IFDs in critically ill patients. 

A possible, important limitation of the present work is the end of study search in 2018, which may have precluded inclusion of some more recent eligible manuscripts. However, the study period was in line with the project protocol, and an updated search for all domains (IA, IC, PJP, and other IFDs) will be run just before the drafting of recommendations, in order not to miss any possible more recent eligible information.

In conclusion, according to our systematic review, there are insufficient solid data for directly evaluating the performance of existing definitions and laboratory tests for the diagnosis of PJP and other non-IA, non-IC IFDs in HIV-negative, non-hematological, non-SOT critically ill adult patients.

## Figures and Tables

**Table 1:** Studies assessing the performance of PCR for the diagnosis of PJP in critically ill patients.

Study, Year [Reference] (Test vs. Reference)	Design	Reference Categories *N*/Total (Prevalence)	Sensitivity	Specificity	PPV	NPV	LR+	LR−	DOR	Population
Azoulay et al., 2009 ^1^ [12] (PCR vs. conventional stains on BALF or sputum)	Cohort Prospective Single -center	Pos/Neg 39/448 patients (8.7%)	87.2% (IS plus BALF) 84% (BALF)	92.2% (IS plus BALF) 93% (BALF)	51.5% (IS plus BALF) 53.1% (BALF)	98.7% (IS plus BALF) 87.2% (BALF)	11 (IS plus BALF) 12 (BALF)	0.14 (IS plus BALF) 0.17 (BALF)	NR	Non-HIV immunocompromised patients admitted to the ICU or pulmonology wards with pulmonary infiltrates and respiratory failure
Rohner et al., 2009 ^2^ [13] (PCR vs. microscopy on BALF)	Cross-sectional Retrospective Single -center	Pos/Neg 33/186 samples (17.7%)	100% (BALF)	92.4% (BALF)	63.4% (BALF)	100% (BALF)	13.5 (BALF)	0 (BALF)	NR	Patients had signs and symptoms and/or radiological abnormalities that included PJP as the differential diagnosis. Mixed patient cohort, including ICU

BALF, broncho-alveolar lavage fluid; DOR, diagnostic odds ratio; HIV, human immunodeficiency virus; ICU, intensive care unit; IS, induced sputum; LR, likelihood ratio; *N*, number of positive cases; Neg, negative; NPV, negative predicted value; NR, not retrievable; PCR, polymerase chain reaction; Pos, positive; PJP, *Pneumocystis jirovecii* pneumonia; PPV, positive predicted value. ^1^ Data reported from the whole cohort of samples (BAL + sputum) and from BALs only; ^2^ data reported for the PCR using Kex-1 primer.

## Data Availability

The data presented in this study are available on reasonable request from the corresponding author.

## Supplementary Materials

The following are available online at https://www.mdpi.com/2309-608X/7/3/176/s1: Search strings.
