# Supplementary material for: Performance of Existing Definitions and Tests for the Diagnosis of Invasive Fungal Diseases other than Invasive Candidiasis and Invasive Aspergillosis in Critically Ill, Adult Patients: A Systematic Review with Qualitative Evidence Synthesis
Source: J Fungi (Basel). 2021 Feb 28;7(3):176. doi: 10.3390/jof7030176 (PMC7997529; doi:10.3390/jof7030176)
Supplement: Supplementary file 1 [file jof-07-00176-s001.zip › Supplementary material.pdf]

Supplementary material

Search strings

Search 1

Pubmed
(“fungemia”[Mesh:Noexp] OR “Invasive Fungal Infections” OR “Invasive Fungal Infection" OR “Pneumonia, Pneumocystis" [Mesh] OR Pneumocyst* OR "Pneumocystis Pneumonia" OR "Pneumocystis Pneumonias") AND (definition* OR criter* OR guideline* OR Recommendation*)

CINAHL
("fungemia"[Mesh:Noexp] OR “Invasive Fungal Infection” OR “Invasive Fungal Infections” OR "Pneumonia, Pneumocystis" [Mesh] OR Pneumocyst* OR "Pneumocystis Pneumonia" OR "Pneumocystis Pneumonias") AND (definition* OR criter* OR guideline* OR Recommendation*)

Cochrane
("fungemia"[Mesh:Noexp] OR “Invasive Fungal Infection” OR “Invasive Fungal Infections” OR "Pneumonia, Pneumocystis" [Mesh] OR Pneumocyst* OR "Pneumocystis Pneumonia" OR "Pneumocystis Pneumonias") AND (definition* OR criter* OR guideline* OR Recommendation*)

Embase
('fungemia'/de OR 'Invasive Fungal Infection*':ti, ab, kw OR 'pneumocystosis'/exp OR Pneumocyst*:ti, ab, kw OR 'Pneumocystis Pneumonia*':ti, ab, kw) AND (definition*:ti, ab, kw OR criter*:ti, ab, kw OR guideline*:ti, ab, kw OR Recommendation*:ti, ab, kw)

Search 2

PubMed
("fungemia"[Mesh:Noexp] OR “Invasive Fungal Infection” OR “Invasive Fungal Infections” OR "Cryptococcosis"[Mesh] OR "Histoplasmosis"[Mesh] OR "Zygomycosis"[Mesh] OR "Hyalohyphomycosis"[Mesh] OR Cryptococc* OR Zygomycos* OR Mucor* OR Histoplasm* OR Fusari* OR "Scedosporium"[Mesh] OR Scedospor* OR Hyalohyphomycosis) AND (definition* OR criter* OR guideline* OR Recommendation*)

CINAHL
("fungemia"[Mesh:Noexp] OR “Invasive Fungal Infection” OR “Invasive Fungal Infections” OR "Cryptococcosis"[Mesh] OR "Histoplasmosis"[Mesh] OR "Zygomycosis"[Mesh] OR Cryptococc* OR Zygomycos* OR Mucor* OR Histoplasm* OR Fusari* OR Scedospor* OR hyalohyphomycos*) AND (definition* OR criter* OR guideline* OR Recommendation*)

Cochrane
("fungemia"[Mesh:Noexp] OR “Invasive Fungal Infection” OR “Invasive Fungal Infections” OR "Cryptococcosis"[Mesh] OR "Histoplasmosis"[Mesh] OR "Zygomycosis"[Mesh] OR "Hyalohyphomycosis"[Mesh] OR Cryptococc* OR Zygomycos* OR Mucor* OR Histoplasm* OR Fusari* OR Scedospor* OR "Scedosporium"[Mesh]) AND (definition* OR criter* OR guideline* OR Recommendation*)

Embase
('fungemia'/de OR 'Invasive Fungal Infection*':ti, ab, kw OR 'cryptococcosis'/mj OR 'histoplasmosis'/mj OR 'zygomycosis'/mj OR 'mucormycosis'/mj OR 'hyalohyphomycosis'/mj OR cryptococc*:ti, ab, kw OR zygomycos*:ti, ab, kw OR mucor*:ti, ab, kw OR histoplasm*:ti, ab, kw OR Fusari*:ti, ab, kw OR Scedospor*:ti, ab, kw OR 'Scedosporium'/mj) AND (definition*:ti, ab, kw OR criter*:ti, ab, kw OR guideline*:ti, ab, kw OR recommendation*:ti, ab, kw)
